# Continuous Cuffless Blood Pressure Estimation Using a Multimodal Conv1D-LSTM Architecture and the SOUNDI^®^ Wearable Device

**DOI:** 10.3390/bios16080412

**Published:** 2026-07-30

**Authors:** Dario Bovio, Alice Turrini, Alice Prandoni, Alberto Porro, Pietro Cerveri, Caterina Salito

**Affiliations:** 1 Biocubica Srl, 20154 Milan, Italy; dario.bovio@biocubica.it (D.B.); alice.prandoni@biocubica.it (A.P.); alberto.porro@biocubica.it (A.P.); 2 Department of Electronics, Information and Bioengineering, Politecnico di Milano, 20133 Milan, Italy; alice.turrini@mail.polimi.it (A.T.); pietro.cerveri@unipv.it (P.C.); 3 Department of Industrial, and Information Engineering, University of Pavia, 27100 Pavia, Italy

**Keywords:** cuffless, wearable, cardiovascular, blood pressure monitoring, deep convolutional networks, multivariate signals, LSTM neural network

## Abstract

Arterial blood pressure (BP) is a crucial physiological parameter reflecting cardiovascular function and exhibiting dynamic oscillations in response to physiological, psychological, and environmental changes. Since conventional cuff-based measurements provide only intermittent “snapshot” readings, there is growing interest in reliable cuffless and noninvasive systems capable of capturing BP dynamics over time. This work proposes a continuous cuffless BP estimation system based on SOUNDI^®^, a proprietary wearable device for multimodal physiological and environmental monitoring developed by Biocubica srl. An end-to-end neural network architecture combining convolutional and long short-term memory (LSTM) layers was designed to automatically extract features from pre-processed signals and directly predict systolic and diastolic BP (SBP and DBP). Data were collected from 20 healthy subjects (7 females, 13 males) using a tailored acquisition protocol with reference targets acquired through the validated GIMA^®^ ABPM50 device. Model performance was evaluated using both a Leave-One-Subject-Out (LOSO) cross-validation framework to assess subject-independent generalizability and a conventional 80/20 segment-level split reflecting a personalized monitoring setup. Under the subject-independent LOSO cross-validation, the model achieved a best-subject MAE of 4.93 mmHg for SBP and 3.76 mmHg for DBP, demonstrating the feasibility of generalizing across unseen individuals despite inter-subject physiological variability. Under the conventional 80/20 split, the framework achieved an MAE of 2.98 mmHg for SBP and 2.68 mmHg for DBP, with RMSE values of 4.16 mmHg and 3.72 mmHg, respectively, and Pearson correlation coefficients of R = 0.95 for SBP and R = 0.94 for DBP. These results establish a promising feasibility proof-of-concept for multimodal cuffless BP tracking under dynamic states and nocturnal conditions, highlighting both the strength of multi-sensor fusion and the value of lightweight calibration for real-world deployment on larger, clinical cohorts.

## 1. Introduction

Continuous and non-invasive blood pressure (BP) monitoring is a cornerstone of modern cardiovascular medicine, critical for managing hypertension and preventing acute adverse events. Since Stephen Hales first demonstrated the invasive measurement of arterial pressure in 1733, the field has evolved toward minimizing patient discomfort and maximizing clinical utility. While traditional intermittent cuff-based sphygmomanometry remains the clinical standard, it lacks the temporal resolution required to capture transient hemodynamic fluctuations, nocturnal dipping patterns, or acute hypertensive responses during daily activities [1,2,3,4,5,6,7].

To overcome these limitations, substantial research has focused on cuffless, continuous BP estimation techniques using wearable physiological sensors. The most common paradigms rely on Pulse Arrival Time (PAT) or Pulse Transit Time (PTT), typically derived from a combination of Electrocardiography (ECG) and Photoplethysmography (PPG). Despite their conceptual simplicity, single- or dual-sensor modalities suffer from severe limitations that hinder their clinical translation and deployment in dynamic ambulatory environments [7,8,9].

First, PAT-based models are inherently unstable under dynamic conditions because vascular tone is non-linearly modulated by both smooth muscle regulation and autonomic nervous system activity, causing the mathematical relationship between transit time and arterial pressure to decouple [10]. Second, peripheral optical signals like PPG are highly susceptible to motion artifacts and localized contact pressure changes, which frequently result in signal saturation, loss of physiological pulse morphology, or severe estimation drift over time [2]. Finally, these localized optical approaches lack the necessary systemic context to differentiate between true blood pressure changes and regional vasomotor responses induced by external environmental stimuli. Consequently, there is an urgent need for robust, multimodal approaches that capture the multi-faceted nature of cardiovascular mechanics [11].

To address this gap, this work introduces a comprehensive multimodal framework utilizing the SOUNDI^®^ system to monitor continuous blood pressure [12]. By simultaneously capturing electrical, mechanical, and environmental domains, including Thoracic Bioimpedance (BIOZ), Phonocardiography (PCG), Ballistocardiography (BCG), Seismocardiography (SCG), and ambient temperature, the system provides a holistically constrained representation of the cardiac cycle. The integration of mechanical chest signals yields direct correlates of cardiac contractility and valvular events, while bioimpedance tracks central fluid shifts and systemic changes, thereby mitigating the vulnerability of localized peripheral sensors to motion and calibration drift [13,14,15,16]. Furthermore, incorporating ambient temperature provides essential context-aware mapping, allowing the estimation framework to account for external thermal vasoconstriction or vasodilation pathways [17].

Processing such a high-dimensional, heterogeneous physiological dataset requires analytical architectures capable of extracting complex, multi-scale dependencies. Traditional linear regression and heuristic machine learning models struggle to capture the non-linear, time-varying interactions inherent in multimodal cardiovascular data [10,18]. Therefore, this study leverages a deep learning architecture combining One-Dimensional Convolutional Neural Networks (Conv1D) and Long Short-Term Memory (LSTM) blocks [19]. Conv1D layers are uniquely suited for physiological signal processing as they automatically extract localized morphological features and shift-invariant patterns across diverse sensor channels without requiring manual feature engineering [18,19]. These spatial features are subsequently processed by LSTM blocks, which excel at capturing the long-term temporal dependencies and sequential dynamics inherent in continuous physiological tracking [11,19]. By mapping these fused spatial–temporal representations directly to systolic and diastolic pressure references, the proposed deep learning framework offers a robust, multi-parameter alternative for continuous, cuffless blood pressure monitoring.

To rigorously evaluate the proposed framework, two distinct validation strategies were implemented: a Leave-One-Subject-Out (LOSO) cross-validation framework to serve as the primary benchmark for subject-independent generalizability, and a conventional segment-level 80/20 split to evaluate model capacity under a calibrated, user-specific setup. This study is presented as a feasibility proof-of-concept for multimodal wearable tracking under dynamic physical and nocturnal conditions.

## 2. Materials and Methods

### 2.1. Data Collection and Acquisition Protocols

Data were acquired from 20 healthy subjects (7 females and 13 males), aged between 21 and 72 years. While a cohort of twenty subjects represents a pilot-scale sample size, this configuration is justified by the richness of the experimental protocol and the resulting data density. Rather than focusing on a large population under static conditions, the primary objective of this work is to evaluate the feasibility of continuous, multi-parameter monitoring under highly dynamic physiological states. Each participant underwent an intensive multi-stage protocol, including postural transitions, cold pressor tests, and full-night sleep tracking, which yielded thousands of continuous cardiac cycles per subject. Consequently, although the subject count is limited, the total number of processed 10-beat instances exceeds several thousand, providing a highly rich and varied dataset with sufficient input variance for pilot-level deep learning feature extraction. This study is therefore presented as a feasibility proof-of-concept, and the cohort size is acknowledged as a key limitation.

The dataset consisted of physiological signals acquired by SOUNDI^®^ (Biocubica srl, Milano, Italy) [12], paired with target systolic blood pressure (SBP) and diastolic blood pressure (DBP) values obtained using a validated cuff-based device.

The acquisition protocol was designed to capture natural physiological blood pressur (BP) variations. To ensure a broad and heterogeneous distribution of target BP values without the administration of vasoactive drugs, the study cohort incorporated a diverse demographic profile in terms of age and gender. Furthermore, BP variability was actively elicited through three distinct methodologies. First, postural changes were utilized by collecting data across standing, sitting, and supine positions. Second, a cold pressor test was performed by immersing the subject’s right hand in cold water to induce a sympathetic vasoconstrictive response [20,21,22]. Finally, nocturnal monitoring was leveraged to exploit spontaneous intra-subject BP oscillations during sleep, thereby capturing physiological modulation in the absence of external stimuli [9].

The first protocol consisted of a 1-h session comprising three 15-min intervals corresponding to standing, lying, and sitting positions, during which the subject was instructed to implement the selected strategies aimed at inducing physiological changes in blood pressure. During each interval, reference blood pressure measurements were acquired every 5 min. In the second phase of the protocol, the subject immersed the right hand in an ice bath (4–6 °C) for one minute, while the cuff was positioned on the left arm; the reference measurement was taken 90 s after hand removal from the water, and a final measurement was acquired after an additional 5 min [20,21]. The second protocol consisted of an overnight recording session during sleep.

**Figure 1:**
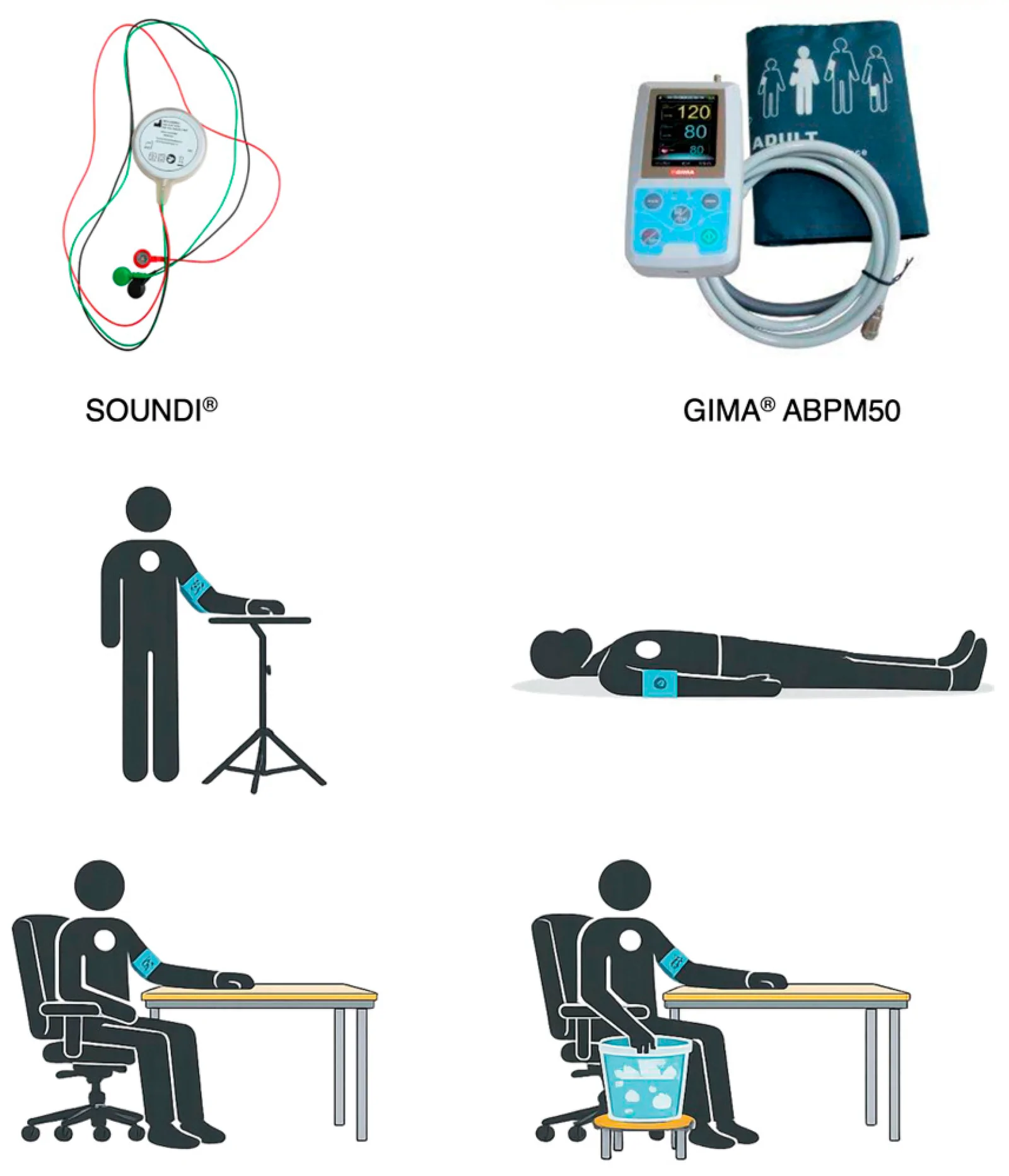
Setup of the simultaneous acquisition of physiological signals and target BP with the two devices.

Figure 1 shows the different protocol setups.

The materials used for data acquisition included SOUNDI^®^ [12], employed to acquire physiological signals, and the GIMA^®^ ABPM50 (GIMA Spa, Milano, Italy) device, used as a validated cuff-based gold-standard reference for determining target BP values.

According to the GIMA^®^ ABPM50 instructions and general medical best-practice guidelines, the cuff must be positioned at heart level when performing the measurement. This requirement was fulfilled in the lying position by keeping the arm alongside the body, in the sitting position by adjusting chair height relative to the desk and subject stature, and in the standing position by using a height-adjustable support to maintain the cuff at the appropriate level.

Reference measurements during nocturnal monitoring were programmed at 15-min intervals.

Data were collected under the Ethical Committee approval of Opinion 3/2019, Politecnico di Milano University.

### 2.2. Data Preparation and Pre-Processing

For both daytime and nighttime protocols, blood pressure was considered approximately constant within a 2-min interval. This assumption allows short-term BP variability to be captured, excluding only beat-to-beat fluctuations. Consequently, each BP reference value was assumed to represent at least the 2-min interval preceding the measurement. Conversely, physiological signals recorded immediately after cuff inflation are affected by a transient rebound effect induced by arterial occlusion; therefore, they were excluded from the dataset.

SOUNDI^®^ data were acquired at a sampling frequency of 400 Hz and downloaded using the dedicated proprietary software [12]. Each acquisition generated a single .dat file containing the recorded raw signals.

Starting from this file, all signals were aligned via linear interpolation to ensure uniform sampling and validity before entering subsequent pre-processing steps.

This workflow resulted in the generation of a single .csv file containing all signals extracted from each SOUNDI^®^ acquisition.

Subsequently, a signal-specific cleaning process based on frequency-domain filtering was applied. Frequency ranges were initially defined according to evidence reported in the literature and further refined through qualitative inspection of the processed signals.

Based on the state of the art in cuffless blood pressure estimation, an initial selection of candidate signals to include in the dataset was defined, while the final configuration was determined empirically through a trial-and-error process guided by qualitative inspection of model outputs. The selected signals were phonocardiogram (PCG), single-lead electrocardiogram (ECG), photoplethysmogram (PPG), thoracic electrical bioimpedance (measured using the same electrodes employed for ECG acquisition and placed at the upper right and lower left thoracic regions), body temperature, and environmental temperature.

In addition, two derived signals were included based on their reported correlation with blood pressure values [13]. Specifically, ballistocardiogram (BCG) and seismocardiogram (SCG) signals, representing body vibrations induced by cardiac activity, were computed by deriving the resultant acceleration vector from the three axial components acquired by SOUNDI^®^. Band-pass filtering was then applied according to definitions reported in the literature (0.5–10 Hz for BCG and 0.6–20 Hz for SCG) [13].

Regarding the filtering of directly acquired SOUNDI^®^ signals, a second-order Butterworth band-pass filter with cut-off frequencies of 30–190 Hz was applied to the PCG signal. For the ECG signal, a second-order Butterworth band-pass filter with cut-off frequencies of 1–80 Hz was applied, attenuating respiratory motion and high-frequency noise due to electrode coupling artifacts and cable movement.

The PPG signal was filtered using a second-order Butterworth band-pass filter in the 0.8–10 Hz range. Lastly, thoracic bioimpedance was processed using a second-order Butterworth high-pass filter with a cut-off frequency of 1 Hz to minimize components related to lung inflation and deflation while preserving cardiac-related information. All the signals selected to feed the model and cut-off frequencies implemented in the filtering step are summarized in Table 1. The GIMA^®^ ABPM50 device was used to acquire reference blood pressure values, which served as target variables in the dataset.

A dedicated Excel workbook was created to store GIMA^®^ ABPM50 acquired values in a structured manner, adopting an acquisition-specific worksheet organization to facilitate automated retrieval in the processing pipeline through the corresponding acquisition ID.

During the daytime acquisition protocol, a GIMA^®^ ABPM50 device was manually activated at the end of each 5-min interval. For nighttime recordings, the device was initiated simultaneously with SOUNDI^®^ and operated in ABPM mode. Reference BP values were automatically recorded at 15-min intervals and stored in the device’s memory. These data were subsequently downloaded as .awp files using the dedicated software, decoded, and automatically used to populate the corresponding acquisition worksheet.

### 2.3. Dataset Construction and Validation Split

After proper preparation of both SOUNDI^®^ and GIMA^®^ ABPM50 acquired data through the described filtering and preprocessing procedures, the dataset was constructed, creating a specific structure according to the steps outlined below, which were applied to each file obtained from a single acquisition.

First, the 2-min segments were extracted. Depending on the acquisition protocol, a 5-min sliding window for the daytime protocol and a 15-min sliding window for the nighttime protocol were applied to the SOUNDI^®^ recording to identify all the samples temporally corresponding to the GIMA^®^ ABPM50 measures. Each of these samples was considered as the final sample of a segment, and the sequence of samples preceding it, corresponding to a temporal duration of 2 min, was extracted as valid data and added to the dataset by considering corresponding samples on all the selected signals in parallel.

A quality check step was incorporated at this stage by setting experience-based amplitude thresholds to crop signal segments affected by excessive noise sometimes caused by motion artifacts. This allowed for the prevention of biased normalization, which could result in excessive attenuation of the signal amplitude when fed into the model. For each acquisition, the specific protocol settings were specified, and, in the case of nighttime recordings, the first valid GIMA^®^ ABPM50 reference measurement was explicitly indicated to ensure correct temporal alignment of the data.

For each of the extracted segments, the ECG signal was processed using the ecg_peaks function from the neurokit2 library to identify all R-peaks occurring within the segment and to be able to subsequently separate individual cardiac beats during later processing steps. The output was obtained as a list of indexes corresponding to the samples at which R-peaks were detected. The list was then associated with the corresponding segment by storing it in an additional field named R_peaks.

The target variables were associated with the respective segment by accessing the Excel worksheet corresponding to the acquisition under analysis and storing the SBP and DBP values in additional dedicated fields of the instance structure. This step also included an integrity check aimed at identifying any NaN values, either in the signal fields or in the target fields, and discarding the entire segment accordingly. NaN values in the target fields were typically associated with missing measurements due to multiple failed attempts of the GIMA^®^ ABPM50 device operating in ABPM mode, which occur because of incorrect subject positioning during sleep. NaN values in the signal fields, instead, were usually due to SOUNDI^®^ recordings extending beyond the monitoring duration of the GIMA^®^ ABPM50 device or to SOUNDI^®^ data being discarded because of poor signal quality.

At this point, an additional empty field named LEN was added to the data structure to reserve space for storing additional information retrieved later in the pipeline.

All individual acquisition files were processed through the described pipeline and saved separately in a local folder. The final dataset was composed of instances consisting of blocks of 10 consecutive beats [18]. One beat was defined as the interval of samples between two consecutive R-peaks considered across all signal fields in parallel. The construction of the dataset object was accomplished by loading the processed acquisition files one by one by means of a for loop, within which a custom function loads pre-processed acquisition dictionaries from a folder, standardizes their beat lengths to a specific number of samples using either resampling or padding, and skips non-signal metadata fields.

The pipeline extracts individual beats from 2-min segments based on R-peaks, records their original lengths, discards abnormally long beats (missed detections), and resamples the valid ones to a uniform size.

**Figure 2:**
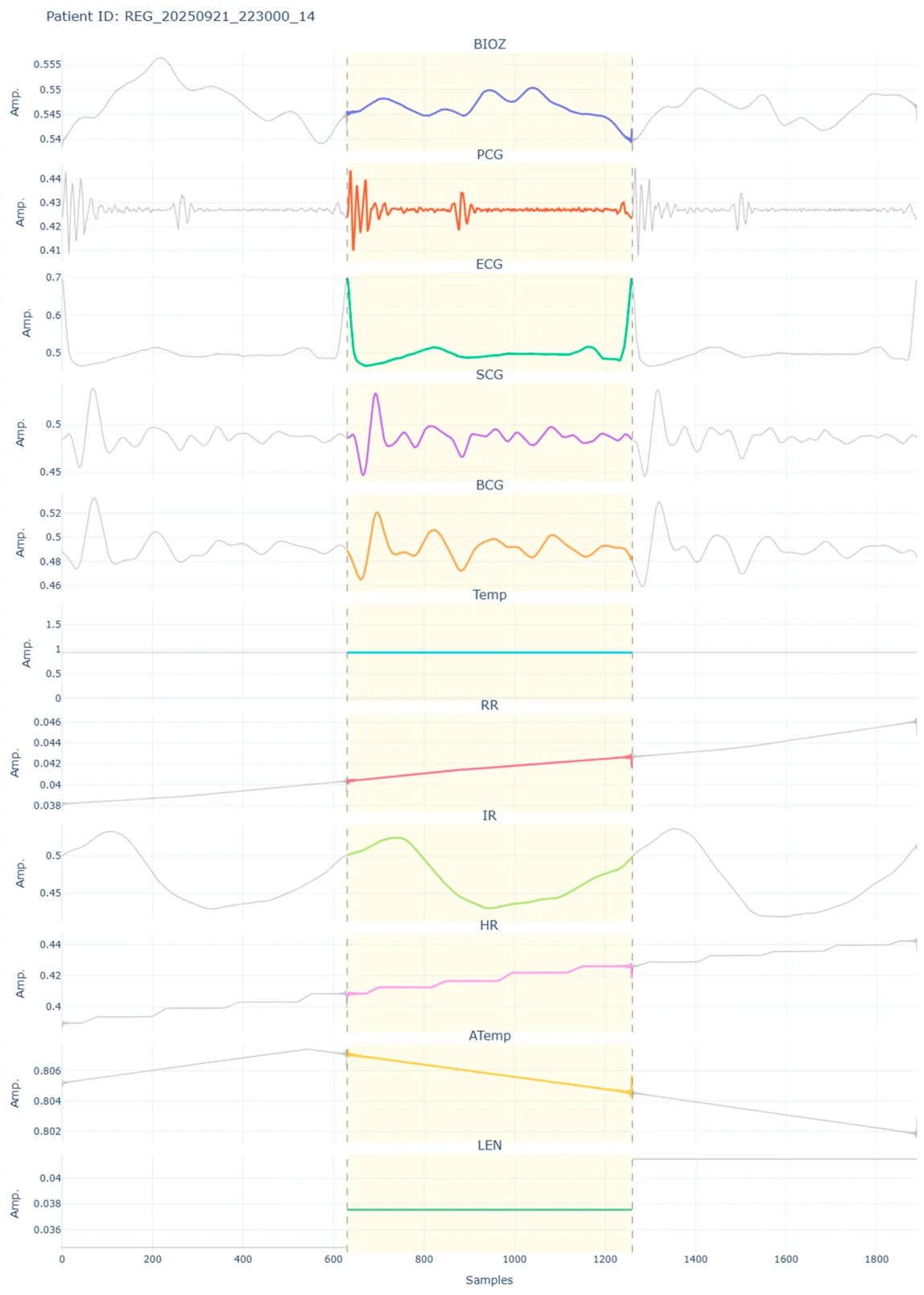
Stacked visualization of three consecutive beats from the multichannel input data. Each row represents one input channel, while the *x*-axis reports the sample index across three concatenated beats. The central beat is highlighted (yellow area) to indicate the segment used as the reference beat, whereas the preceding and following beats provide local temporal context. This representation allows visual inspection of the morphology and relative amplitude variations across all input signals.

Figure 2 represents the final data structure divided into beats.

The selected beat length was determined by considering a minimum heart rate of 40 bpm, corresponding to approximately 600 samples per beat. Based on empirical observations from the acquired data, this value was extended to 630 samples per beat in order to accommodate the longest beats observed, which exceeded 600 samples in some low heart rate acquisitions.

To ensure that each 10-beat block was composed exclusively of beats belonging to the same 2-min interval, a buffer-based strategy was implemented to progressively populate the final data structure. More specifically, the logic groups every 10 consecutive beats from the same segment into a single data instance using a fixed-size buffer. Incomplete groups at the end of a segment are discarded to maintain perfect temporal alignment.

To evaluate the robustness and generalizability of the proposed predictive framework under a conventional subject-independent training protocol, the overall dataset was partitioned using an 80/20 train–test split. The splitting criteria ensured that segments from the same subject did not cross-contaminate the training and testing subsets. Furthermore, a validation subset (representing 15% of the training partition) was utilized during the training phase to monitor learning progress, guide hyperparameter tuning, and prevent overfitting.

### 2.4. Network Architecture and Implementation

The proposed predictive model was developed in Python (version 3.12.9) using the TensorFlow/Keras framework within the Visual Studio Code IDE (verison 1.94.1). To bypass the limitations of handcrafted feature extraction, an end-to-end learning strategy was adopted. The network automatically processes raw, multi-channel physical waveforms, extracting local features via one-dimensional convolutional (Conv1D) blocks and modeling long-term temporal dependencies through a Long Short-Term Memory (LSTM) layer [18].

**Figure 3:**
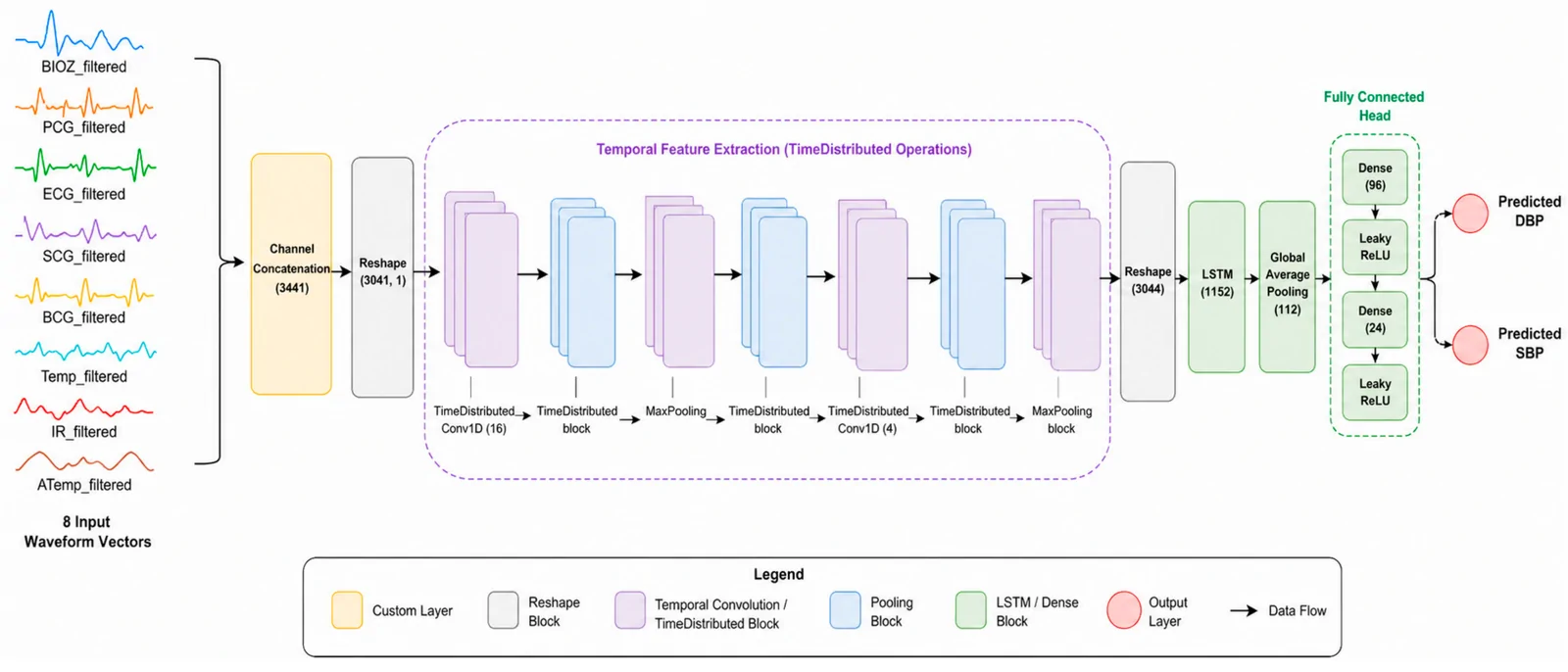
Schematic representation of the proposed multimodal deep learning architecture. Eight input waveform (ECG, PPG, PCG, BCG (derived), SCG (derived), Thoracic Bioimpedance, Body Temperature, and Environmental Temperature) vectors are first combined through a custom concatenation layer and reshaped before entering a temporal feature extraction module based on TimeDistributed convolutional and pooling operations. The extracted temporal features are then processed by an LSTM layer, followed by global average pooling and a fully connected head. The network produces two separate outputs corresponding to the predicted diastolic blood pressure (DBP) and systolic blood pressure (SBP).

The input instance has an inherently three-dimensional structure corresponding to: (i) the sequence of 10 beats, (ii) the temporal resolution of 630 samples per beat, and (iii) the selected input signal channels. To apply convolutional and pooling operations independently to each individual beat, the Conv1D and MaxPooling layers are enclosed in Keras TimeDistributed wrappers. Figure 3 explains the entire structure of the network.

The model maps these features to systolic (SBP) and diastolic (DBP) blood pressure values through a fully connected regressor head. The final network structure encompasses 1.48 million trainable parameters. The complete architectural configuration is detailed in Table 2.

To enhance network training, specific design choices were integrated:

- Activation Functions: Intermediate convolutional and dense layers utilize LeakyReLU. Preliminary trials with standard ReLU introduced a gradient saturation bias, upper-bounding the predicted blood pressure values. LeakyReLU maintains a small non-zero slope for negative inputs, mitigating this effect.
- Custom Epsilon-Insensitive Loss: To account for the intrinsic ±8 mmHg measurement tolerance of the GIMA® ABPM50 reference device, a custom loss function was defined with *ϵ* = 8. Prediction-target discrepancies below this threshold are not penalized, preventing the model from overfitting to the measurement noise of the reference device.
- Feature Selection and Alignment: Immediately after the input layer, a custom layer organizes the multi-channel signals following the approach of Tanveer et al. ([11]). Single cardiac beats are segmented, resampled to 630 samples, concatenated, and appended with the normalized original beat length as an additional feature before feeding the subsequent convolutional blocks.

### 2.5. Model Training and Optimization Protocol

Model optimization was performed using the Adam optimizer, which was selected following preliminary comparative experiments against Root Mean Square Propagation (RMSProp). Hyperparameters, including the number of filters, kernel sizes, LSTM units, and dense neurons, were initially bounded via manual exploratory phases and subsequently optimized using an automated Keras tuning procedure.

The training process was configured with an initial learning rate ($\alpha =10^{-3}$). To stabilize gradient descent, mitigate overfitting, and guarantee convergence, three specific callback mechanisms were integrated directly into the training pipeline:

- ModelCheckpoint: Monitored validation loss to automatically track and save only the optimal weight configurations that represented the best generalizability.
- ReduceLROnPlateau: Dynamically scaled down the learning rate by a factor of 0.2 if the validation loss failed to improve for 10 consecutive epochs, establishing a minimum learning rate threshold at 10^−5^.
- EarlyStopping: Enforced the strict termination of the training process if no validation loss reduction was registered for 30 consecutive epochs (patience = 30).

### 2.6. Statistical Analysis

To quantitatively assess and compare the performance of the proposed deep learning framework in predicting systolic (SBP) and diastolic (DBP) blood pressure, three standard statistical evaluation metrics were utilized: Mean Absolute Error (MAE), Root Mean Squared Error (RMSE), and the Pearson Correlation Coefficient (R). Additionally, predictive performance was visually evaluated using Bland–Altman analysis to assess systematic bias and limits of agreement (LOA) between the predicted estimations and GIMA^®^ ABPM50 reference measurements.

All the statistical analyses were conducted via SigmaPlot (version 13) software or Python (version 3.12.9). A *p*-value < 0.05 was considered to indicate statistical significance.

## 3. Results

### 3.1. Conventional Training

**Figure 4:**
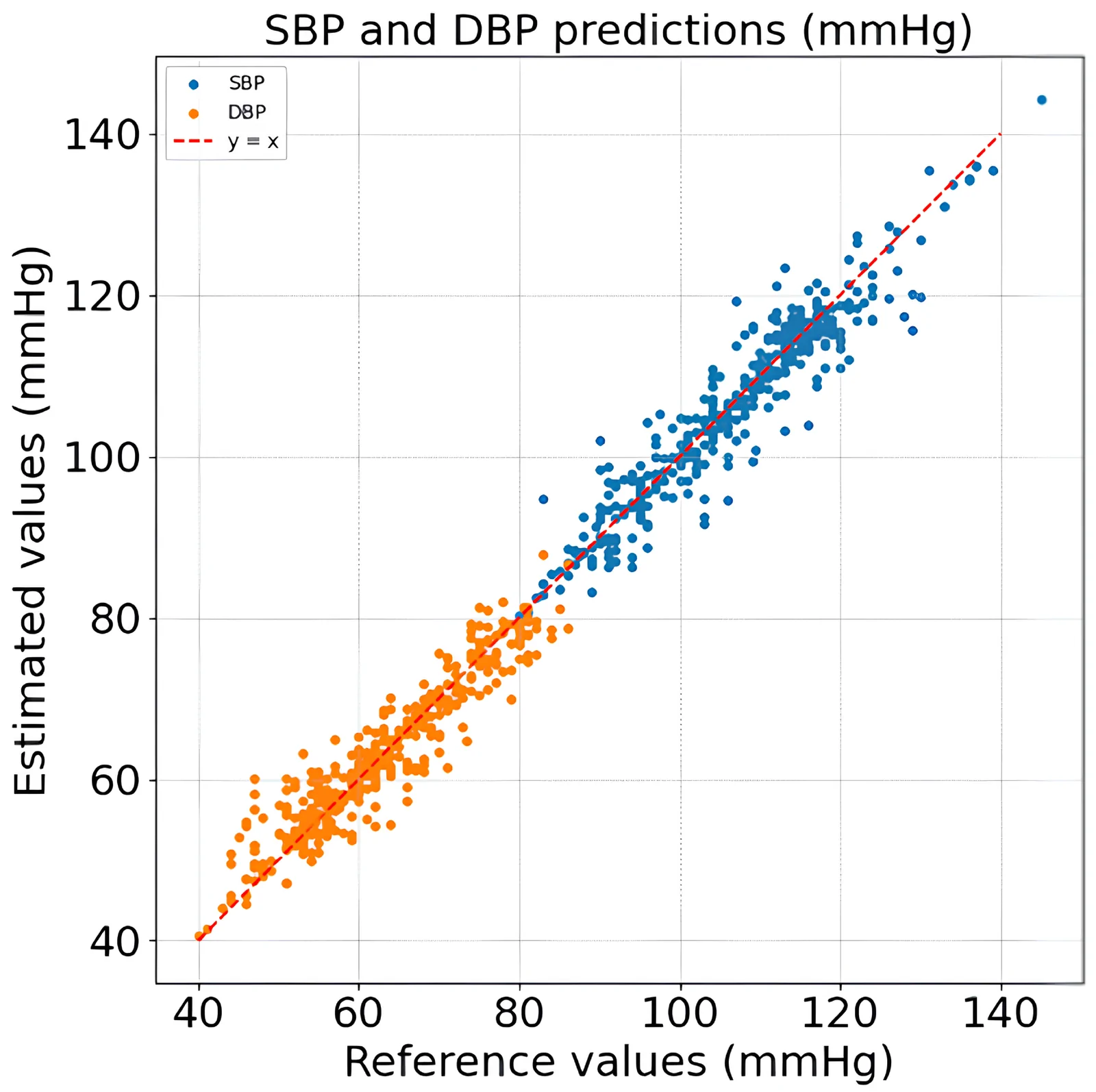
Relationship between reference and estimated values of BP.

Test-set performance was assessed both quantitatively and visually. Figure 4 illustrates the correlation plots for both systolic and diastolic blood pressure estimations. The proposed deep learning framework demonstrated limited dispersion, indicating a strong correlation between predicted and reference values (R = 0.95 for SBP and R = 0.94 for DBP).

**Figure 5:**
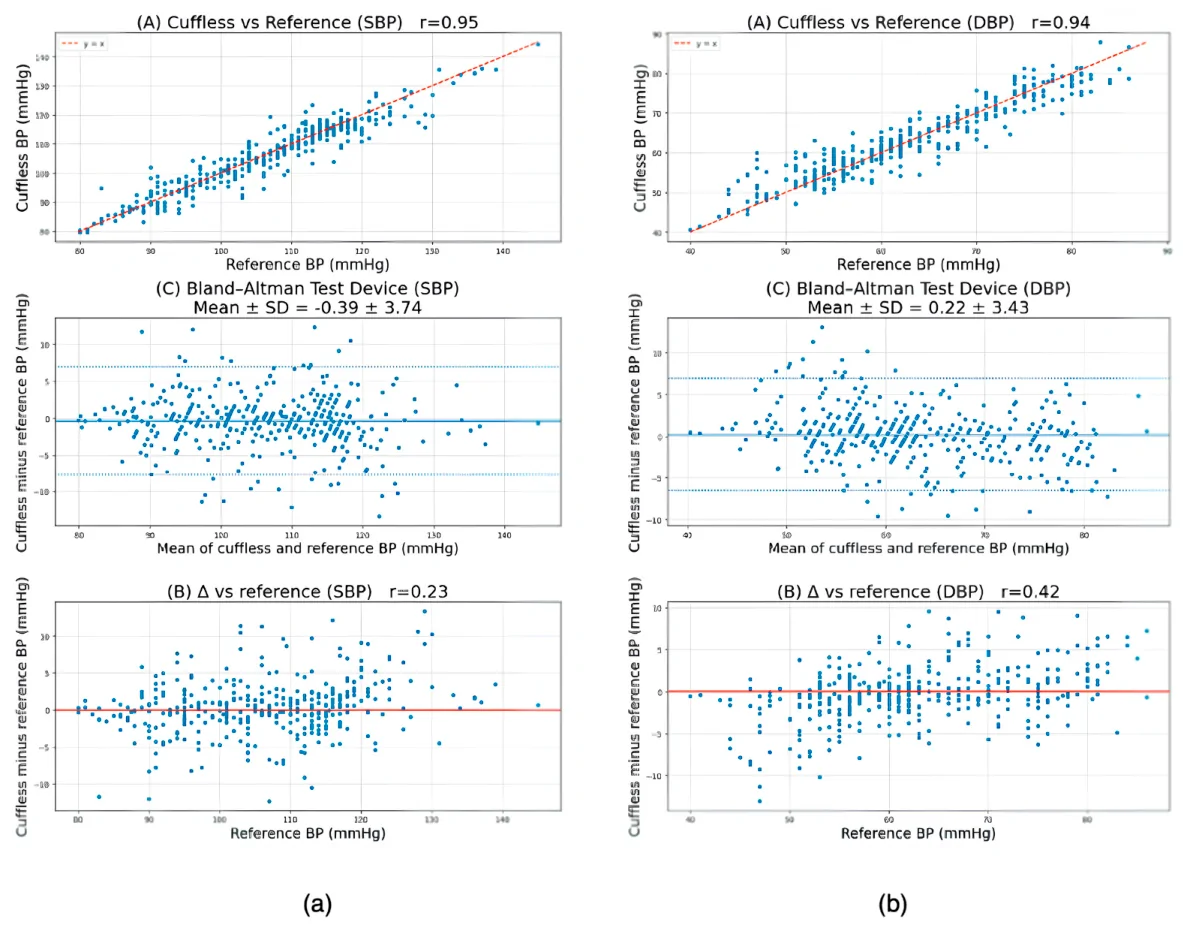
(**a**) Scatter plots of SBP predicted vs. reference values, Bland–Altman analysis and prediction errors vs. reference value (from top to bottom). (**b**) Scatter plots of DBP predicted vs. reference values, Bland–Altman analysis and prediction errors vs. reference value (from top to bottom).

Further evidence of model robustness was provided by the Bland–Altman analysis and by the scatter plot of the prediction errors (predicted minus reference values) versus the corresponding reference values. In both cases, the points were distributed around zero without evident systematic trends, supporting the absence of major bias across the explored blood pressure range (Figure 5a,b).

### 3.2. Leave-One-Subject-Out

**Figure 6:**
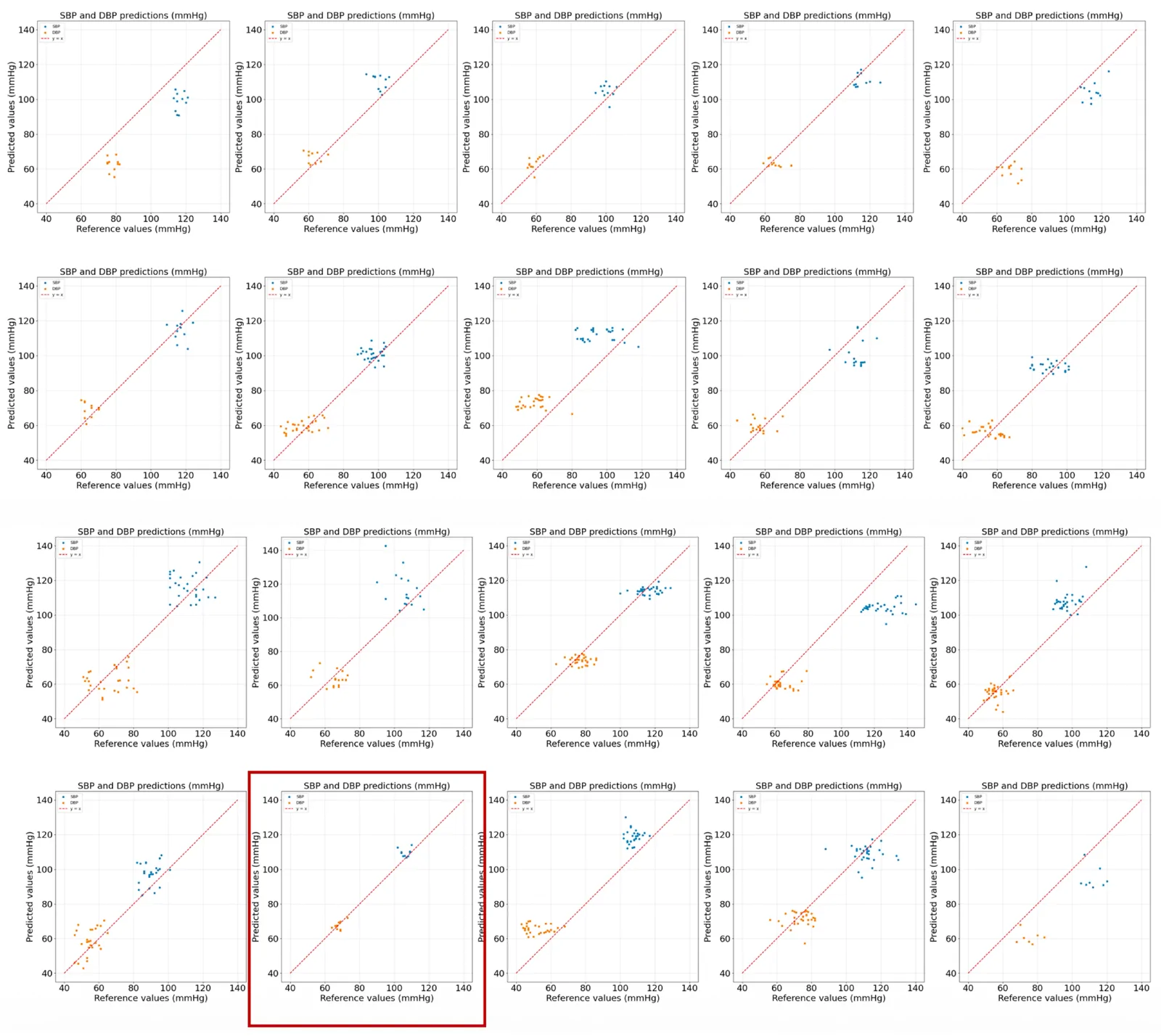
Scatter plots of predicted vs. reference values of each of the 20 folds implemented in the LOSO training strategy. The subject highlighted in red represents the best prediction.

To more rigorously assess the generalization capability of the proposed model, a leave-one-subject-out (LOSO) training strategy was implemented. The subject-wise split was enabled by using the unique identifier associated with each acquisition. At each iteration, all instances belonging to one subject were systematically excluded from the training set and used exclusively as the test set for the trained model. Within each LOSO iteration, the training–validation split was performed, adopting a 90%/10% proportion to obtain a validation set size comparable to that of the test set. Prediction performances on the test set were assessed visually by means of the scatter plot of predicted vs. real values reported in Figure 6 and numerically by calculating MAE and RMSE (Table 3).

The best results were obtained from subject 17, for which the model predicted BP values with an MAE of 4.92 mmHg for SBP and 3.76 mmHg for DBP, and an RMSE of 6.29 mmHg and 5.01 mmHg for SBP and DBP, respectively.

### 3.3. Ablation Study and Signal Contribution Analysis

To evaluate the relative importance of the integrated physiological domains and to justify the selection of the final 8-channel multimodal input, an ablation study was performed (Table 4). The optimal configuration was refined through an empirical trial-and-error process, comparing various signal combinations, ranging from a minimal electro-mechanical set to an extended multi-parameter suite. This analysis aimed to identify the most informative modalities for mapping the non-linear interactions of the cardiovascular system while ensuring systemic robustness against motion artifacts and calibration drift.

The results of the ablation study highlight that while PPG plays a dominant role in blood pressure estimation, yielding the lowest numerical errors in early trials, it performs best when backed by a wider sensor array. We ultimately chose the 8-signal framework because mechanical signals like BCG and SCG provide a holistic view of the cardiac cycle, capturing heart contractility and aortic ejection that peripheral sensors alone cannot detect.

Additionally, bioimpedance (BIOZ) acts as a stabilizer against sensor drift by tracking central fluid shifts, while ambient temperature provides the necessary context to account for environmental factors like vasoconstriction. By fusing these diverse inputs, the system becomes significantly more robust, overcoming the typical vulnerabilities of localized sensors to ensure clinical-grade accuracy.

## 4. Discussion

The development of continuous, non-invasive, and cuffless blood pressure (BP) estimation algorithms represents a fundamental shift in cardiovascular monitoring, moving away from isolated intermittent measurements toward a continuous evaluation of blood pressure variability (BPV). In this study, the implementation of an end-to-end Conv1D-LSTM neural network architecture was directly informed by two distinct methodologies in the literature, which provided a benchmark for feature extraction strategies: the waveform-based deep learning approach by Tanveer et al. [11] and the handcrafted feature-engineering paradigm proposed by Kachuee et al. [10].

Tanveer et al. [11] demonstrated the efficacy of minimizing manual feature engineering by using a hybrid architecture—combining a hidden artificial neural network (ANN) layer with stacked long short-term memory (LSTM) blocks—to directly process raw, concatenated electrocardiogram (ECG) and photoplethysmogram (PPG) waveforms. Their model leveraged clinical data from 39 highly heterogeneous Intensive Care Unit (ICU) patients from the MIMIC I database, underscoring the performance advantages of deep recurrent frameworks when exposed to rich temporal structures. Conversely, Kachuee et al. [10] focused on continuous BP estimation by extracting explicit physiological and morphological descriptors, primarily Pulse Arrival Time (PAT) and its derivative parameters, from the MIMIC II database. Although they implemented Principal Component Analysis (PCA) to reduce 190 engineered variables down to 15 principal components while preserving 98% of the signal energy, their reliance on explicit timing mechanisms showcased the strict limitations of traditional machine learning (e.g., AdaBoost regression) when mapping highly non-linear physiological interactions.

The performance achieved by the proposed Conv1D-LSTM model demonstrates the high accuracy of combining multidimensional physiological and environmental data, yielding a Mean Absolute Error (MAE) of 2.98 mmHg for systolic blood pressure (SBP) and 2.68 mmHg for diastolic blood pressure (DBP) under a conventional 80%/20% data split. When evaluated against the state-of-the-art models, our architecture reveals crucial physiological and algorithmic insights. Compared to the hierarchical model of Tanveer et al. [11], our framework exhibits slightly higher error metrics (MAE of 2.98 mmHg vs. 1.10 mmHg for SBP). This divergence is largely attributable to the clinical profile and volume of the underlying datasets; while Tanveer et al. utilized an intensive care cohort characterized by severe acute hemodynamical variations and a significantly larger number of samples, our dataset comprises 20 healthy subjects undergoing controlled physical stimuli (postural changes, cold pressor tests, and nocturnal dipping during sleep). However, the proposed deep learning framework demonstrates a lower estimation error compared to the handcrafted feature regression approach proposed by Kachuee et al. [10], which reported a substantially higher SBP estimation error (MAE of 11.17 mmHg). This performance improvement highlights the capability of end-to-end temporal architectures to automatically extract salient morphological features from physiological waveforms, bypassing the limitations and information loss inherent to manual fiducial point extraction.

This stark contrast heavily validates our core architectural thesis: the automatic feature extraction inherent in deep learning structures, specifically the temporal modeling capability of Conv1D layers coupled with the long-range dependency tracking of LSTM units, is profoundly more robust than traditional manual feature engineering. Handcrafted parameters like PAT are highly sensitive to sensor placement, arterial stiffness, and physiological noise, whereas an end-to-end network autonomously learns complex latent representations directly from the multi-channel time series.

When evaluating the performance of the proposed framework against the state of the art, a fundamental methodological distinction must be highlighted. The comparison of absolute estimation metrics (such as MAE) across different publications does not represent a direct, head-to-head competitive benchmark. This lack of direct comparability stems from critical discrepancies in dataset characteristics, subject demographics, and clinical environments.

Specifically, many prominent studies in the cuffless blood pressure estimation literature leverage large-scale clinical databases containing ICU patient data (such as the MIMIC database), which are characterized by high-frequency invasive arterial blood pressure lines and specific pathological hemodynamic states. Conversely, our study relies on an ambulatory, pilot-scale cohort of healthy subjects subjected to active physical stressors and multi-hour nocturnal sleep monitoring.

Therefore, the comparisons summarized in Table 5 are presented purely to position our methodological choices, sensing modalities, and preliminary pilot-level results within the wider landscape of cuffless estimation literature, rather than as a direct competitive benchmark under identical testing conditions.

Furthermore, as summarized in Table 5, the proposed framework integrates an array of eight distinct physical and environmental signals acquired from the SOUNDI^®^ system, including thoracic bioimpedance, phonocardiogram, derived ballistocardiogram, seismocardiogram, and ambient parameters. This multi-sensor approach yields Pearson correlation coefficients of R = 0.95 for SBP and R = 0.94 for DBP, accompanied by narrow Bland–Altman Limits of Agreement. Rather than replacing a larger clinical cohort, this multi-sensor fusion strategy enriches the pilot-scale dataset by injecting highly correlated spatial, electrical, and mechanical dimensions of the cardiovascular cycle directly into the latent feature space, thereby partially mitigating the limited cohort size through increased information density per subject.

In this context, the inclusion of environmental temperature as an input feature plays a critical role in optimizing the accuracy of blood pressure estimation. Environmental temperature exerts a direct, well-documented physiological effect on the human cardiovascular system through vasomotor responses. Exposure to colder ambient temperatures triggers sympathetic activation and peripheral vasoconstriction to conserve core body heat, which in turn increases peripheral vascular resistance and elevates systemic arterial blood pressure. Conversely, warmer environments promote vasodilation, resulting in a physiological decrease in blood pressure.

By incorporating ambient temperature data directly into the multi-sensor input space, the neural network is provided with essential environmental context. This context-aware mapping is highly valuable for resolving physiological ambiguities in the physical waveforms. For instance, a localized reduction in photoplethysmogram (PPG) amplitude can be caused either by thermal vasoconstriction due to cold ambient air or by vasoconstriction induced by an acute psychological stress response. Equipping the model with environmental context enables the latent layers to correctly differentiate and weigh these distinct mechanisms, leading to more robust and generalized blood pressure estimations.

A central finding of this study lies in the performance disparity between the Leave-One-Subject-Out (LOSO) cross-validation and the conventional segment-level 80/20 split.

The conventional split yields remarkably low error metrics (MAE < 3 mmHg), primarily because randomly partitioning 10-beat segments from the same continuous recording allows the network to memorize subject-specific physiological baselines and anatomical coupling features. While this setup demonstrates high efficacy for personalized or pre-calibrated monitoring regimes, it does not reflect performance on unseen individuals due to intra-subject correlation and temporal autocorrelation.

Conversely, the LOSO evaluation isolates entire subjects, testing true inter-subject generalizability. Under LOSO, the estimation error increases to an MAE of 10.57 mmHg for SBP and 8.01 mmHg for DBP.

This performance gap highlights the impact of inter-individual variability, such as differences in baseline vascular compliance, body composition, and autonomic regulation, on biomechanical waveforms. While the current LOSO metrics fall short of strict uncalibrated clinical validation standards, they establish a solid baseline for pilot-scale multi-sensor wearable technology, pointing toward the necessity of domain adaptation or lightweight initial calibration in real-world clinical deployment.

Despite the promising performance and high correlation obtained by the proposed multi-sensor framework, several limitations must be explicitly acknowledged.

First and foremost, the pilot-scale cohort is limited to twenty healthy subjects. While the rich acquisition protocol yielded a high density of continuous cardiac cycles per subject to train the deep learning model, the clinical implications of relying on a small, healthy cohort are significant:

- Lack of Vascular Pathologies: Since the dataset consists entirely of healthy individuals, the predictive model remains completely untested on clinical populations presenting with cardiovascular pathologies. Specifically, in patients with clinical hypertension, arterial stiffness, or calcified vascular walls, the physiological coupling between biomechanical waveforms (such as those captured by phonocardiography, seismocardiography, and ballistocardiography) and actual arterial pressure changes is known to be severely altered. Consequently, the features learned by the model in this study might not translate to pathological hemodynamics, and further validation on diverse patient populations is strictly required.
- Overfitting and Demographic Generalizability: Although subject-independent validation (80/20 train–test split) was implemented to prevent data leakage, a small cohort size inherently increases the risk that the neural network overfits to the specific demographic and physiological distribution of our pilot group. The underlying physiological signatures of twenty individuals, even when captured across dynamic activities and deep sleep, cannot represent the vast heterogeneity of the general population in terms of vascular compliance, body mass index, and autonomic regulation.

Future clinical studies will therefore focus on expanding the cohort size and actively enrolling individuals with diagnosed cardiovascular conditions to assess the robustness, generalizability, and clinical utility of the SOUNDI^®^-based monitoring system.

Additionally, incorporating domain adaptation techniques or transfer learning paradigms could allow the model to pre-train on large public databases (such as MIMIC) and subsequently fine-tune on the unique multimodal sensor array of the SOUNDI^®^ device, thereby optimizing inter-subject generalizability without losing the specialized context of our proprietary hardware.

## 5. Conclusions

This study provides a preliminary proof-of-concept of an end-to-end multimodal deep learning framework for continuous, cuffless blood pressure estimation using the proprietary SOUNDI^®^ wearable device. By leveraging a Conv1D-LSTM architecture, the system demonstrated robust predictive accuracy under conventional validation splitting, achieving Mean Absolute Error values for both systolic and diastolic blood pressure that fall well within the clinical precision tolerances specified by standard ambulatory protocols. Notably, the autonomous extraction of localized spatiotemporal features from raw physiological and environmental waveforms proved significantly superior to traditional handcrafted paradigms, effectively capturing complex, non-linear cardiovascular adaptations without manual engineering bias.

Although subject-independent cross-validation through the Leave-One-Subject-Out strategy highlighted expected challenges due to inter-subject morphological variability, the framework establishes a baseline for scalable non-invasive monitoring. The high correlation indicators and balanced error distributions reinforce the potential of utilizing multi-channel signal combinations, including thoracic bioimpedance, phonocardiogram, and environmental variables, to model systemic vascular dynamics. Future translational efforts will focus on expanding the clinical cohort to encompass highly diverse vascular topologies and integrating domain adaptation paradigms. This will pave the way for true generalized transfer learning architectures, transforming the SOUNDI^®^ platform into a continuous, unobtrusive monitoring solution across ambient, homecare, and clinical sleep applications.

## Figures and Tables

**Table 1:** The table summarizes all the signals selected to feed the model with the respective pre-processing steps applied.

Signal	Pre-Processing	Cut-Off Frequency
PCG	Linear interpolation + Zero-phase BP Butterworth	30–190 Hz
PPG	Linear interpolation + Zero-phase BP Butterworth	0.8–10 Hz
ECG	Linear interpolation + Zero-phase BP Butterworth	1–80 Hz
BIOZ	Linear interpolation + Zero-phase Butterworth HPF + Savitzky–Golay (4th order)	1 Hz
BCG	Linear interpolation + Zero-phase BP Butterworth	0.5–10 Hz
SCG	Linear interpolation + Zero-phase BP Butterworth	0.6–20 Hz
body temperature	Linear interpolation	-
environmental temp	Linear interpolation	-

**Table 2:** Structural organization, output dimensions, and hyperparameter configurations of the network layers.

Layer Name/Type	Type of Operation	Key Hyperparameters	Output Shape/Units	Activation Function
Input Layer	Custom Concatenation	Signal Segment Selection	$\left(10,3041,1\right)$	None
TimeDistributed Conv1D (1)	Temporal Feature Extraction	Kernel Size = 3, Filters = 16	$\left(10,3039,16\right)$	LeakyReLU
TimeDistributed Conv1D (2)	Temporal Feature Extraction	Kernel Size = 3, Filters = 32	$\left(10,3037,32\right)$	LeakyReLU
TimeDistributed MaxPooling	Dimensionality Reduction	Pool Size = 2	$\left(10,1518,32\right)$	None
TimeDistributed Dropout	Regularization	Dropout Rate = 0.2	$\left(10,1518,32\right)$	None
Reshape Layer	Tensor Flattening	Dimension Alignment	$\left(10,48576\right)$	None
LSTM	Temporal Sequence Modeling	Units = 64	64	Tanh (Default)
Dense (1)	Fully Connected Mapping	Units = 96	96	LeakyReLU
Dense (2)	Fully Connected Mapping	Units = 24	24	LeakyReLU
Output Layer	Linear Regression	Units = 2	2 (SBP, DBP)	Linear

**Table 3:** Subject-specific and cohort-wide blood pressure estimation errors (all values in mmHg).

Subject	SBP MAE	SBP RMSE	DBP MAE	DBP RMSE
1	16.70	17.60	15.21	16.05
2	9.37	11.45	5.27	6.65
3	6.56	7.62	5.47	6.51
4	6.50	8.33	6.34	7.62
5	10.18	11.54	9.51	11.75
6	6.14	7.95	5.40	7.01
7	7.02	9.11	6.59	7.95
8	17.68	19.54	15.41	16.44
9	13.73	15.25	6.44	8.34
10	7.90	9.56	8.01	9.22
11	10.42	12.55	9.36	11.48
12	12.69	17.83	7.30	8.75
13	5.35	6.75	5.25	6.62
14	20.55	22.53	5.50	7.32
15	10.93	12.38	4.40	5.87
16	8.84	10.45	6.84	8.41
17	4.93	6.29	3.76	5.01
18	11.68	13.12	14.14	15.89
19	6.94	9.72	6.00	7.55
20	17.25	19.21	13.92	15.44
Mean	10.57	12.44	8.01	9.49
SD	4.57	4.73	3.71	3.69

Note: SBP = Systolic Blood Pressure; DBP = Diastolic Blood Pressure; MAE = Mean Absolute Error; RMSE = Root Mean Squared Error; SD = Standard Deviation.

**Table 4:** Summary of signal configurations and corresponding estimation errors (MAE/RMSE) obtained during the empirical selection process (80/20 split).

Configuration	SBP MAE/RMSE (mmHg)	DBP MAE/RMSE (mmHg)
ECG + PPG (Baseline)	3.10/4.46	2.62/3.66
ECG + PPG + Mechanical (PCG, SCG, BCG)	2.87/4.09	2.49/3.49
Full Multimodal (Proposed)	2.98/4.16	2.68/3.72

**Table 5:** The table summarizes the comparison between this study and the approaches developed by Kachuee et al. ([10]) and Tanveer et al. ([11]).

Study	Dataset	Cuffless Solution	Prediction Error (mmHg)	Bland–Altman & Pearson (r)
Md. Sayed Tanveer et al. [11]	Data from 39 patients in ICU belonging to different age groups and sex.	ECG and PPG signals processed through a hierarchical ANN-LSTM model.	SBP: MAE = 1.10, RMSE = 1.56 DBP: MAE = 0.58 RMSE = 0.85	LOA *: SBP: [−3.08, +3.03] DBP: [−1.67, +1.65] Correlation (r): SBP: r = 0.99 DBP: r = 0.99
Mohammad Kachuee et al. [10]	Physionet’s MIMIC II (version 3, accessed on September 2015) online waveform database (about 1000 unique subjects).	Feature extraction from PAT using ECG and PPG signals processed through an ML regression model (AdaBoost)	SBP: MAE = 11.17 DBP: MAE = 5.35	Correlation (r): SBP: r = 0.59 DBP: r = 0.48
This work	Data from 20 healthy subjects.	SOUNDI^®^ acquired signals (including physiological and environmental) processed through a CONV1D-LSTM model	SBP: MAE = 2.98 RMSE = 4.16 DBP: MAE = 2.68 RMSE = 3.72	LOA: SBP: [−7.72, +6.94] DBP: [−6.50, +6.94] Correlation (r): SBP: r = 0.95 DBP: r = 0.94

* Limits of Agreement.

## Data Availability

The raw data supporting the conclusions of this article will be made available by the authors on request.

## Abbreviations

The following abbreviations are used in this manuscript:

Abbreviation	Full Name
ABP	Arterial Blood Pressure
ABPM	Ambulatory Blood Pressure Monitoring
ANN	Artificial Neural Network
API	Application Programming Interface
BCG	Ballistocardiogram
BIOZ	Thoracic Electrical Bioimpedance
BP	Blood Pressure
BPV	Blood Pressure Variability
Conv1D	One-Dimensional Convolutional
DBP	Diastolic Blood Pressure
ECG	Electrocardiogram
ICU	Intensive Care Unit
IDE	Integrated Development Environment
LeakyReLU	Leaky Rectified Linear Unit
LOA	Limits of Agreement
LOSO	Leave-One-Subject-Out
LSTM	Long Short-Term Memory
MAE	Mean Absolute Error
ML	Machine Learning
NaN	Not a Number
PAT	Pulse Arrival Time
PCA	Principal Component Analysis
PCG	Phonocardiogram
PPG	Photoplethysmogram
ReLU	Rectified Linear Unit
RMSE	Root Mean Squared Error
SBP	Systolic Blood Pressure
SCG	Seismocardiogram
SD	Standard Deviation
